# Removal of organotin compounds and metals from Swedish marine sediment using Fenton’s reagent and electrochemical treatment

**DOI:** 10.1007/s11356-021-17554-8

**Published:** 2022-01-05

**Authors:** Anna Norén, Célia Lointier, Oskar Modin, Ann-Margret Strömvall, Sebastien Rauch, Yvonne Andersson-Sköld, Karin Karlfeldt Fedje

**Affiliations:** 1 Department of Architecture and Civil Engineering, Division of Water Environment Technology, Chalmers University of Technology, 412 96 Gothenburg, Sweden; 2 Recycling and Waste Management, Renova AB, Box 156, 401 22 Gothenburg, Sweden; 3 Swedish National Road and Transport Research Institute (VTI), Box 8072, 402 78 Gothenburg, Sweden; 4 Department of Architecture and Civil Engineering, Division of Geology and Geotechnics, Chalmers University of Technology, 412 96 Gothenburg, Sweden

**Keywords:** Tributyltin (TBT), Metals, Electrolysis, Chemical oxidation, Sediment management, Remediation

## Abstract

Metal and tributyltin (TBT) contaminated sediments are problematic for sediment managers and the environment. This study is the first to compare Fenton’s reagent and electrochemical treatment as remediation methods for the removal of TBT and metals using laboratory-scale experiments on contaminated dredged sediment. The costs and the applicability of the developed methods were also compared and discussed. Both methods removed > 98% TBT from TBT-spiked sediment samples, while Fenton’s reagent removed 64% of the TBT and electrolysis 58% of the TBT from non-spiked samples. TBT in water phase was effectively degraded in both experiments on spiked water and in leachates during the treatment of the sediment. Positive correlations were observed between TBT removal and the added amount of hydrogen peroxide and current density. Both methods removed metals from the sediment, but Fenton’s reagent was identified as the most potent option for effective removal of both metals and TBT, especially from highly metal-contaminated sediment. However, due to risks associated with the required chemicals and low pH level in the sediment residue following the Fenton treatment, electrochemical treatment could be a more sustainable option for treating larger quantities of contaminated sediment.

**Supplementary Information:**

The online version contains supplementary material available at 10.1007/s11356-021-17554-8.

## Introduction

Organotin compounds, especially tributyltin (TBT), used to be of high importance as an antifouling agent in the maritime industry (Ayanda et al., 2012). Between the 1950s and 1980s, the man-made product TBT was used at a large scale as the main active biocide in antifouling paints (Du et al., 2014). However, the toxic effects of TBT were identified, and the compound was revealed to be an endocrine disruptor and have a similar toxicity to the better known polychlorinated dibenzodioxin compounds (Sousa et al., 2013). TBT bioaccumulates and causes deformities and death of biota even at concentrations as low as 0.001–0.5 μg/L (U’Ren, 1983; Gibbs et al., 1991). The TBT degradation products, dibutyltin (DBT), and monobutyltin (MBT), also have toxic properties but to a lesser extent, in the order of MBT < DBT < TBT (Hoch, 2001; Ayanda et al., 2012). In Europe, the use of TBT in antifouling paint was banned for application to small ships in the late 1980s, and to large vessels in 2003 (EU Regulation (EC) No 782/2003 and Directive 89/677/EEC). Despite the ban, persistent TBT is still present in the environment, and in dark and anoxic conditions, such as in sediments at greater depths, the reported half-life spans from 10 to 90 years (Dowson et al., 1996; Viglino et al., 2004). TBT’s persistence and toxicity are not only problematic from an environmental perspective, but also for sediment managers such as port owners, who regularly need to dredge the sediment to maintain the water depth. Depending on the TBT content, sediment type, and local regulations, both reuse and disposal can be difficult, while a content reduction could enable more management options (Norén et al., 2020).

TBT, C_12_H_28_Sn, is often associated with the presence of boat paint flakes in sediments. The most commonly used TBT derivative in boat paint was TBT oxide (TBTO, (C_12_H_28_Sn)_2_O) (Yebra et al., 2004; Arevalo and Calmano, 2007; Saeki et al., 2007). In seawater, TBT is typically present in three forms: chloride (TBTCl), hydroxide (TBTOH), and carbonate (TBTHCO_3_) (Arevalo and Calmano, 2007). The TBT binds to the fine fractions in the sediment, such as silt, clay, or humic matter (Du et al., 2014), although the sorption of TBT and organotins into sediment particles is reversible. When TBT is left in the sediment, it will leach out over time and spread in the environment. Antizar-Ladislao (2008) identified contaminated sediment as one of the scientific community’s most difficult issues to solve. Consequently, it is a challenge to find a technology that effectively can remove TBT from sediment and preferably degrade it. Thermal treatment, steam stripping, enhanced leaching, biodegradation, and phytoremediation have been proposed as potential methods for the remediation of organotin-contaminated sediments (Du et al., 2014). However, thermal treatment and steam stripping are associated with high energy costs, the biodegradation and phytoremediation are slow, other techniques such as solvent extraction and sorption are more suitable for wastewater treatment (Du et al., 2014), and leaching did not result in complete removal of TBT (Norén et al., 2021). Compared to the other methods, electrochemical and chemical degradation have the potential to rapidly degrade organotins (Ganiyu and Martínez-Huitle, 2021). In general, oxidative techniques such as electrochemical treatment and Fenton’s reagent (the addition of hydrogen peroxide (H_2_O_2_) and ferrous iron (Fe^2+^)) can convert organic pollutants into simple and relatively harmless inorganic compounds through reaction with hydroxyl radicals (HO^•^) (Duarte et al., 2019; Carbajo et al., 2021). Oxidation has been efficient in water treatment, but studies on their efficiency in sediments are limited, and the results showed less effective degradation and more complicated reactions than in water (Brosillon et al., 2014). These oxidative techniques seem to be suitable to be tested for the removal of organotins from contaminated sediment (Eq. 1) and could potentially also degrade other contaminants, e.g., polycyclic aromatic hydrocarbons (PAHs) (Stichnothe et al., 2002, 2005; Arevalo and Calmano, 2007; Ayanda et al., 2012; Pedersen et al., 2017).

1 $${\mathrm{C}}_{12}{\mathrm{H}}_{28}\mathrm{Sn }+ 80 {\mathrm{HO}}^{\bullet }+4{\mathrm{H}}^{+} \to 12{\mathrm{CO}}_{2}+{\mathrm{Sn}}^{4+}+ 56{\mathrm{H}}_{2}\mathrm{O}$$

Chemical oxidation of organotin compounds is dependent on the amounts of hydroxyl radicals that are produced, and for Fenton’s reagent, this depends on the ratio of H_2_O_2_ and Fe^2+^ (Kawahara et al., 1995; Meric et al., 2004; Benatti et al., 2006). The optimal ratios of H_2_O_2_:organic content and H_2_O_2_:Fe^2+^ depend on the compounds to be oxidized, the composition of the matrix, and the sediment and water total organic carbon (TOC) content. Fenton’s reaction may also be naturally formed in soil pores, in the presence of ferrous iron (Ferrarese and Andreottola, 2010).

Electrochemical remediation has been reviewed by Du et al. (2014) and found to be suitable for the treatment of both aqueous and solid phases contaminated with TBT. In addition, this method has shown a capacity to desorb and degrade the chemical pollutants from low porosity geological material, and the efficiency of the electrochemical remediation appears to be inversely proportional to grain size (Acar and Alshawabkeh, 1996; Ferrarese and Andreottola, 2010). This makes it suitable for fine-grained marine sediments. A previous study showed that both boron-doped diamond (BDD) and iridium dioxide catalysts could be used as anodes for the removal of organotin compounds from shipyard wastewater and both hydroxyl radicals and reactive chlorine species generated at the electrodes likely contributed to the removal (Arevalo and Calmano, 2007). However, as organotin and metal removal from water is less complex and more studied than removal from sediment, the removal efficiency may vary greatly between water and sediment using the same method. In advanced electrochemical oxidation processes, pollutants are degraded by direct oxidation at the anode or, indirectly, by oxidants (e.g., ozone, ferrate, chlorine, and peroxo compounds) generated at the anode (Silva et al., 2021). The highest production of hydroxyl radicals has been reported when using BDD anodes (Moreira et al., 2017). The choice of cathode material could also impact the reactions during electrolysis. Titanium (Ti) is a relatively inert metal that has previously been used for the cathodic recovery of metals in aqueous solutions (Modin et al., 2012) and could potentially be efficient for metal recovery in sediments.

The study presented here aims at investigating the removal of TBT and metals from contaminated sediment through oxidation by Fenton’s reagent and electrolysis. To the best of our knowledge, this is the first study that compares Fenton’s reagent and electrochemical oxidation for the removal of TBT and metals in TBT-spiked and naturally contaminated sediments. In addition, the approximate costs and applicability of the techniques were assessed. The results from this study can be useful for stakeholders managing contaminated sediment and enabling the use of treated sediments.

## Materials and methods

### Sampling

**Fig. 1:**
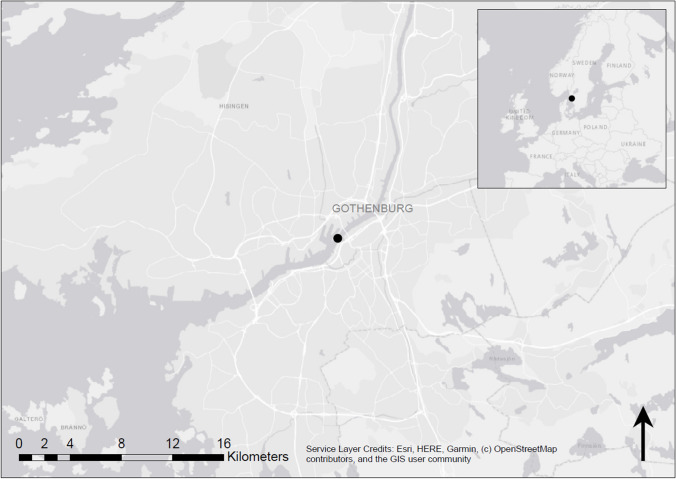
Sediment sample origin in the Göta Älv riverbed in the central part of Gothenburg, Sweden

Sediment was sampled in the Göta Älv riverbed in Gothenburg, Sweden (Fig. 1). The sediment originated from a waterway with boat traffic and is surrounded by trafficked urban areas. About 1 km upstream is an active shipyard known to have high TBT levels in the sediment. TBT is most likely also present in the marina at the riverbank, which is in level with the sampling location. The sediment was collected during a dredging operation, performed by the local port authorities to increase the water depth. The sediment was collected using a grab dredger and transported to land, where it was sampled. In total, ~ 200 L sediment was collected and placed in 40 organotin-free plastic boxes of 5 L each. Larger objects within the sediment, such as mussels, were removed. The sediment in each box was homogenized through mixing and stored at − 22 ℃. Prior to the experiments, the sediment was thawed in a refrigerator at 7 ℃.

### Sample preparation and analysis

The sediment samples collected from the Göta Älv riverbed are referred to as the original sediment. A portion of the sediment was spiked with TBT to achieve a higher TBT concentration of approximately 30 mg/kg, to facilitate the observation of degradation processes and trends. Spiking was performed by adding tributyltin chloride (TBTCl) (96% Sigma-Aldrich) dissolved in 10% methanol and ultra-pure water (Thermo Scientific, 18.2 MΩ cm) to the original sediment. The spiked sediment was then homogenized by mixing for 3 days, followed by evaporation in a 40 ℃ oven for 5 days, and in a 20 ℃ fume hood for 17 days, to ensure the TBT was associated with the sediment and did not remain in the water phase. Spiked and original samples were sent for chemical analysis for the content of TBT, DBT, and MBT.

During the sampling, an ocular inspection was done to characterize the sediment, and the grain size distribution of the sediment was investigated by sieving and sedimentation according to the International Organization for Standardization (ISO) method ISO 11277:2009 (2009). The sediment’s loss on ignition (LOI) and dry weight (DW) were determined using the Swedish Standard Institute’s method SS-EN 028,113 (1981) and total organic content (TOC), according to standardized methods ISO 10694:1995 (International Organization of Standardization 1995), European Standard EN 13,137:2001 (2001), and EN 15,936:2012 (2012).

To investigate the degradation of TBT in water, ultra-pure water (Thermo Scientific, 18.2 MΩ cm) and sea salts (Sigma-Aldrich) were mixed to get two saline waters: slightly saline water (sea salts 2.4 g/L) and saline water (sea salts 35 g/L). TBTCl (96% Sigma-Aldrich) was dissolved in 10% methanol and added to the slightly saline and saline water, and both samples were sent for chemical analysis for concentrations of TBT, DBT, and MBT.

The organotin compounds TBT, DBT, and MBT were analyzed at an external accredited laboratory, according to method ISO 23161:2011 for ﻿sediment (International Organization for Standardization 2011) and method ISO 17353:2004 for spiked water and selected leachates (International Organization for Standardization 2004). The standard method SS-EN ISO 17294–1, 2: 2016 (mod) (Swedish Standard Institute 2016a and b) and U.S EPA-method 200.8:1994. (mod) (U.S EPA 1994) were used for major and minor elements in both sediment and leachates.

### Fenton’s reagent experimental setup

For the Fenton’s reagent oxidation, the effect on removal of TBT, DBT, and MBT was investigated by varying the ratios of hydrogen peroxide (H_2_O_2_) and ferrous iron (II, Fe^2+^) added to spiked and original sediments (Table 1).

**Table 1:** Ratios of H_2_O_2_:TOC and H_2_O_2_:Fe^2+^, and added concentrations of H_2_O_2_ [M] and Fe^2+^ [M] during treatment with Fenton’s reagent. Sample names starting with FS correspond to experiments on spiked sediment and FO correspond to original sediment

Experiment	Ratio^a^ H_2_O_2_:TOC	Ratio^b^ H_2_O_2_:Fe^2+^	[H_2_O_2_] (M)	[Fe^2+^] (M)
FS(0;0)	-	-	0	0
FS(0.01;4)	0.01	4	0.003	0.0007
FS(0.2;7)	0.2	7	0.07	0.01
FS(1;2)	1	2	0.3	0.17
FS(5;2)	5	2	1.7	0.84
FS(5;5)	5	5	1.7	0.33
FS(5;10)	5	10	1.7	0.17
FS(5;15)	5	15	1.7	0.11
FS(5;20)	5	20	1.7	0.08
FS(5;40)	5	40	1.7	0.04
FS(7;5)	7	5	2.3	0.47
FO(5;0)	5	-	1.7	0
FO(5;5)	5	5	1.7	0.33
FO(10;5)	10	5	3.3	0.66

Ferrous sulfate heptahydrate (FeH_14_O_11_S > 99% Sigma-Aldrich) solutions with different concentrations in ultra-pure water (Thermo Scientific, 18.2 MΩ cm), as shown in Table 1, were added to sediments with a corresponding dry weight of 80 g and under mixing. Exposure to light was prevented by covering the setup with aluminum foil. The pH was adjusted to pH 3 using sulfuric acid (H_2_SO_4_, 96%), as the Fenton’s reaction efficiency decreases above pH 3 (Kuo, 1992; Pignatello et al., 2006). Hydrogen peroxide was added as 6 equal aliquots at 20-min intervals to increase the concentrations of hydroxyl radicals (HO^**•**^) and hydroperoxyl radicals (HOO^•^), resulting in a more efficient degradation (Tang and Huang, 1997). The aliquots were added to reach the concentration described in Table 1. After the addition of H_2_O_2_, the volume of the liquids added to the sediment was 0.5 L, resulting in a liquid-to-solid ratio (L/S) of 6.25 L/kg. The sediment slurry was continuously mixed for 23 h and was then allowed to settle for 1 h. The supernatant was centrifuged at 4000 × G for 5 min. Sediment was obtained from the initial setting, but also after centrifugation, and both these sediments were collected and mixed before analysis. The supernatant after centrifugation was collected for further analysis.

### Electrochemical experimental setup

#### Spiked sediment and water

Electrochemical experiments were done in a 1 L glass vessel containing a 5 × 10 cm^2^ niobium anode mesh, coated with boron-doped diamond (Nb/BDD) (~ 120 cm^2^ surface area) (Neocoat, Switzerland) and a 5 × 10 cm^2^ titanium plate cathode (Alfa Aesar). The electrodes faced each other at a 10 cm distance. Dry spiked sediment (80 g) and ultra-pure water were added to the system at an L/S ratio of 6.25. The pH of the system (~ 8.2) was not adjusted. A 30 DC power source (British Standard Tester) was used to control the applied voltage. A digital multimeter was used to measure the current across a 10 Ω resistor. The experimental parameters are shown in Table 2. After each experimental run, the sediment slurry was centrifuged at 4000 × G for 10 min. Sediments and leachates were collected and analyzed separately.

**Table 2:** Time, voltage, and average current for sediment samples during electrochemical treatment. Sample names starting with ES correspond to experiments on spiked sediment and EO correspond to original sediment

Experiment	Time (h)	Voltage (V)	Current (mA)	Sediment (g DW)
ES(5 V)	24	5	30	80
ES(21 V)	24	21	350	80
ES(23 V)	24	23	350	80
EO(0 V)	24	0	0	80
EO(15 V) *	24	15	430	80
EO(19 V)	24	19	710	80
EO(20 V)	24	20	700	80
EO(24 V)	24	24	1100	80
EO(19 V) 400 g	24	19	1100	160
EO(24 V) 400 g	24	24	1500	160

^*^Experiment done in triplicates

The same equipment and experimental setup were used for the TBT spiked 0.5 L saline (35 g/L sea salts, Sigma-Aldrich) and slightly saline water (2.4 g/L sea salts, Sigma-Aldrich) samples; 15 V was used for both the slightly saline and saline water samples. However, due to the difference in conductivity in the samples, different currents were reached (0.02 mA for the slightly saline water and 0.16 mA for the saline water). For each water sample treated, a TBT spiked blank sample was retained, to which no electrical current was applied.

#### Original sediment

Electrochemical experiments on wet original sediment were done in a 1-L glass vessel containing a 10 × 10 cm^2^ niobium anode, coated with boron-doped diamond (Nb/BDD) (Neocoat, Switzerland) and a 10 × 10 cm^2^ titanium plate cathode (Alfa Aesar). The electrodes were 9 cm apart. Sediment corresponding to 80 g DW and ultra-pure water at an L/S ratio of 6.25 was added to the beaker under mixing conditions, resulting in a pH of 8.2 prior to the electrolysis. In two experiments, sediment corresponding to 160 g DW was used (EO(19 V) 400 g and EO(24 V) 400 g). A 30 DC power source (British Standard Tester) was used to control the applied voltage. Applied voltages and currents are shown in Table 2. After each experimental run, the sediments and leachates were collected and analyzed separately as in experiment 1.

## Results and discussion

### Characterization of sediment

The original sediment was characterized as sandy silt, containing gravel, mussels, and shells. Grain size analysis was performed on two samples from different depths, confirming a relatively homogenous sediment, at least for fine particles up to 0.02 mm (Supplementary information Figure A1). The TBT content was ~ 180 times higher in the spiked samples than in the original samples, with corresponding numbers for MBT and DBT of ~ 40 and ~ 70 (Table 3). The significantly higher TBT content in the spiked samples is due to the addition of TBTCl. The higher presence of DBT and MBT in the spiked samples, in comparison to the original sediment, is due to TBT degradation during the preparatory procedure involving mixing, heating, and evaporation procedure. It is also possible that the mixing broke down some of the paint flakes in the sediment, which in combination with the increased oxidative conditions (resulting from the mixing and evaporation procedures) favored TBT degradation. TBT in the spiked sediment was easier to degrade as a large quantity of TBTCl was freely available and less strongly sorbed to sediment particles, which has also been seen in wastewater samples (Arevalo and Calmano, 2007). In a study by Burton et al. (2006), it was seen that in a sediment with higher organic content, ~ 2.6%, there was a positive correlation between how long time the TBT compound had been caught in the sediment and increased difficulty to desorb TBT from the sediment, while in low organic content sediment, ~ 0.5%, the effect of time was low. Here, the sediment had an organic content of 2.2% (Table 3) so the aging may also impact how easily the TBT is removed from the sediment. Thus, TBT may be more easily degradable/leachable in spiked sediment than in the original sediment, where it was introduced through sedimented paint flakes in the form of di(tributyl)tin oxide (DTBTO) and sorption of TBT from surrounding waters pre sampling (Lagerström et al., 2017; Norén et al., 2021). Additionally, DTBTO encapsulated in paint flakes may be less available for degradation and leaching as it may have migrated into the inner pores of the sediments, thereby becoming more strongly bound.

**Table 3:** The average content of TBT, DBT, MBT [µg/kg DW], and Sn [mg/kg DW] in original and spiked sediment, together with standard deviation (STD) and the number of analyzed samples (no. of samples). In addition, the dry weight (DW) [%] and total organic content (TOC) [% DW] are shown

	Original samples			Spiked samples		
	Average	STD	No. of samples	Average	STD	No. of samples
DW [%]	52	4.2	11	97	0.1	3
TOC [% DW]	2.2	0.2	5	2.2	-	1
Organotin compounds [µg/kg DW]						
TBT	160	53	4	25,000	2800	3
DBT	39	11	4	2400	490	3
MBT	19	9.1	4	730	150	3
Metals [mg/kg DW]						
Sn (tot)	3.2	0.57	9	15	0.68	3

The content of TBT and DBT in the original sample, and all organotins in the spiked samples (Table 3), is classified as *very high content*, while the MBT content in the original sample is classified as *high content* according to the Swedish marine sediment classification for the content of organic pollutants (SGU, 2017). The Norwegian environmental quality standards for sediment indicate that extensive acute toxic effects would be found in all three sediments due to the TBT content (Miljødirektoratet, 2016; Direktoratsgruppen vanndirektivet, 2018). This means that the sediment poses a risk to biota in the surrounding environment, which may motivate dredging even though dredging might not be necessary to maintain water depth.

Metal contents were determined in both the original and spiked samples. The only difference was a higher Sn content in the spiked samples, caused by the addition of TBTCl (Table 3). The Cd, Cu, and Hg contents exceeded the background levels but were low enough not to have chronic effects on aquatic organisms in case of long time exposure (Supplementary information Table A1) (Miljødirektoratet, 2016; Direktoratsgruppen vanndirektivet, 2018). According to Canadian guidelines, the Cu and Hg contents were at levels that could cause infrequent effects in aquatic organisms (Canadian Council of Ministers of the Environment n.d.). The rest of the analyzed metals were at background levels or do not have a reference value, see Supplementary information Table A1.

### Organotin removal

#### Fenton’s reagent experiments

**Fig. 2:**
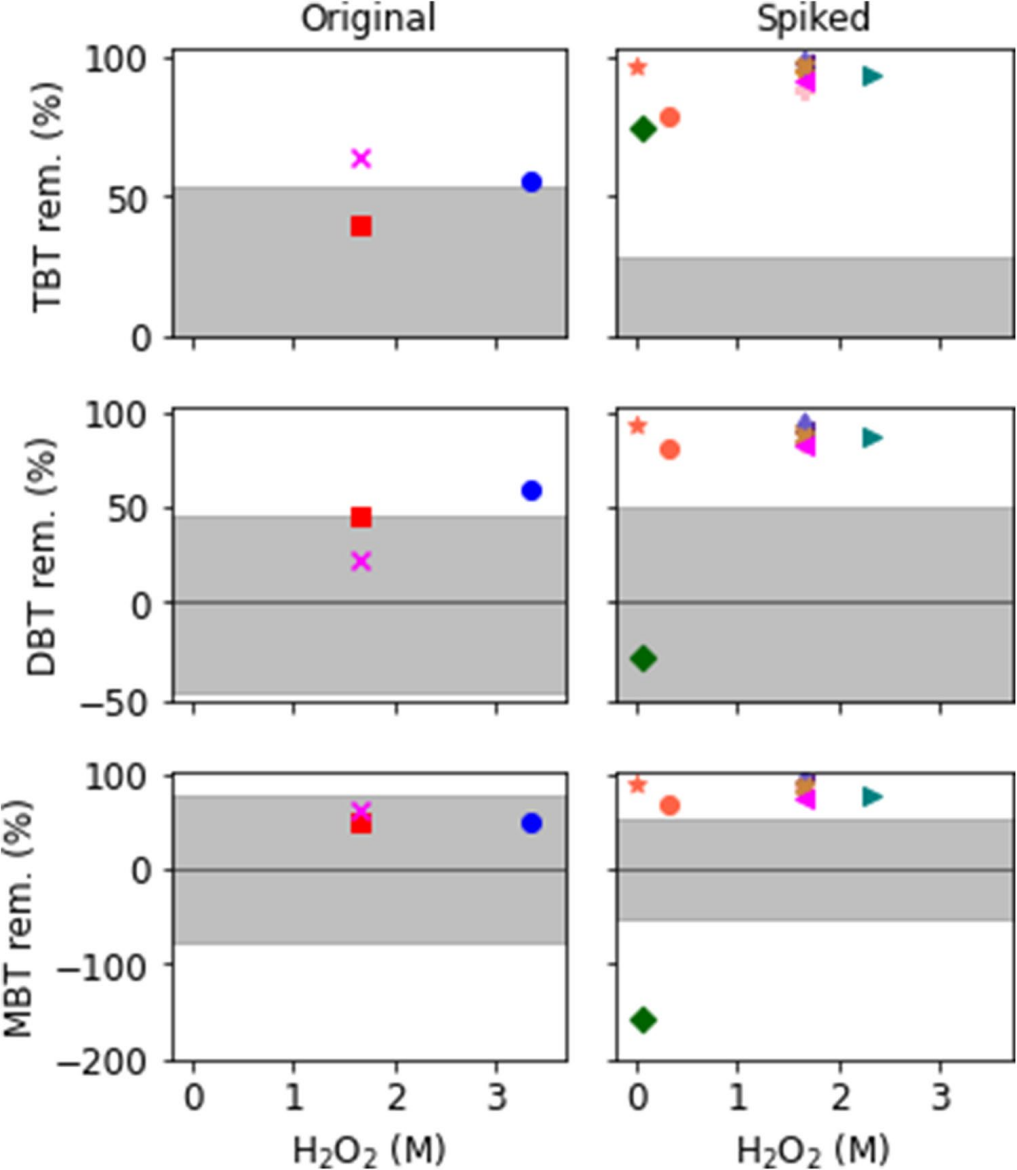
Visualization of the results presented in Table 4. The TBT, DBT, and MBT removals (%) in original (graphs on the left) and spiked sediment (graphs on the right) in relation to used concentrations of hydrogen peroxide (H_2_O_2_). Each experiment is given a unique color and marker to highlight which results (TBT, DBT, and MBT) belong together. Note that where there is more than one point with the same concentration of H_2_O_2_ in the diagram, the Fe^2+^concentrations are different. The shaded areas are marking the interval in which an increase or reduction is significant (p < 0.05, one-sample t test) considering variations in initial content

Fenton’s reagent was used for the oxidation of TBT, DBT, and MBT on spiked (FS, *n* = 12), and original (FO, *n* = 3) sediment. The removal rates for the compounds in all sediments are presented in Table 4 and visualized in Fig. 2. In the figure, the removal of TBT, DBT, and MBT, respectively, is presented in separate graphs. Each experiment has been given a unique color and marker, which is used in all three graphs. The shaded area in each graph represents the range in which a change in contaminant content could be explained by natural deviation in the sediment (*p* < 0.05, one-sample *t* test). Thus, markers that are found outside the gray area represent an experiment in which the content reduction is statistically caused by the treatment procedure. In the original samples, the TBT content was reduced from 160 µg/kg (Table 3) to, at best, 57 µg/kg, i.e., a 64% reduction (Table 4), for sample FO(5;0), to which no Fe^2+^ had been added. In this sample, DBT was reduced by 22% and MBT by 63%. The TBT content in the residual sediments was still classified as *very high content* according to the Swedish marine sediment classification for organic pollutants, and acute toxic effects may still prevail (Miljødirektoratet, 2016; SGU, 2017). MBT was reduced to *medium high content* and DBT remained as *very high content*. The degradation and removal efficiency for TBT in original sediments varied from approximately 40–64%; the corresponding removal from the spiked samples was much more efficient and varied from approximately 52–98% (Fig. 2). The much higher removal rate for the spiked samples may be due to the amount of TBT readily available for oxidation, as discussed in chapter Characterization of sediment (Lagerström et al., 2017; Norén et al., 2021). It is also likely that there is a competition between TBT and other organic compounds in the sediment about the radicals needed for the degradation reaction, while in the spiked sediments, TBT made up a larger proportion of the easily oxidized compounds, and therefore, a larger fraction of TBT was removed.

**Table 4:** The content of TBT, DBT, and MBT [µg/kg DW] in the residual sediment after treatment with Fenton's reagent

Experiment	TBT µg/kg DW	DBT µg/kg DW	MBT µg/kg DW
FS(0;0)	12,000	1700	790
FS(0.01;4)	960	140	76
FS(0.2;7)	6300	3100	1900
FS(1;2)	5300	460	240
FS(5;2)	2100	400	200
FS(5;5)	1900	260	140
FS(5;10)	900	300	94
FS(5;15)	560	160	50
FS(5;20)	2800	390	110
FS(5;40)	700	230	83
FS(7;5)	1600	290	160
FO(5;0)	57	31	7.1
FO(5;5)	95	22	9.4
FO(10;5)	70	16	9.7

Figure 2 presents the removal efficiency for TBT, DBT, and MBT in relation to the amount of H_2_O_2_ added. Note, that although there is more than one point with the same concentration of H_2_O_2_ in Fig. 2, the added amounts of Fe^2+^ are different, see Table 1. The results show that the optimal reduction of TBT, DBT, and MBT in the spiked samples (98%, 94%, and 93%, respectively) is related to sample FS(5;15), which had a relatively high concentration of H_2_O_2_ in the added solution, i.e., H_2_O_2_:TOC = 5 and H_2_O_2_:Fe^2+^ = 15. For the original sample FO(10;5), see Table 4, to which the highest amount of H_2_O_2_ was added, the DBT content was significantly lower than in the other samples. This indicates that a high addition of H_2_O_2_ is efficient for the degradation of TBT and may also be effective for other types of organotin compounds (e.g., phenyltins). However, the optimal H_2_O_2_/Fe^2+^ ratio may vary, as the many competing reactions occurring are also influenced by the surrounding factors. For the spiked samples, 52–98% of the original TBT was removed and no relationship between the removal efficiency and the addition of H_2_O_2_ was observed (Table 4). Potentially, an addition of ~ 1.5 M H_2_O_2_ would reach a high removal rate, without overusing the chemical, as the removal rate after this point did not increase further.

In Eqs. 2–9, some of the most common reactions for Fenton’s reagent are shown (Pignatello et. al., 2006). From the Fenton mechanism, as described in the work by Neyens and Baeyens (2003), it is clear that by increasing the concentration of H_2_O_2_ and/or Fe^2+^ in the reaction solution, the amount of HO^**•**^ is raised to a level known as the optimal concentration. A further increase after this of either Fe^2+^or H_2_O_2_ leads to decreased efficiency of the oxidation system. This can be explained by the ability of H_2_O_2_ or its conjugated base (HO_2_^−^) to act as a HO^**•**^ trap when present in excessive concentrations. Excess Fe^2+^can also react with and capture the produced HO^**•**^ and HOO^**•**^. By performing the experiment at acidic pH (pH ≤ 3), as was done in this study, the formation of Fe(OH)_2_ is reduced, which lowers the radical production (Watts et al., 2002; Pignatello et al., 2006). The formation of radicals and the reaction rate of Fenton’s reaction are affected by pH, and the most optimal catalytic activity is obtained at pH 3 because the concentrations of ferrous (Fe^2+^) and ferric (Fe^3+^) are highest at this pH value (Wang, 2008). If the pH is low, complexes are formed with Fe^2+^ (Lu et al., 2018), which contributes to lower concentrations of Fe^2+^ that can form hydroxyl radicals (HO^•^). At low pH, HO^•^ is also consumed by the reaction with H_3_O^+^ (Tang and Huang, 1997). On the other hand, at high pH, the reaction rate is reduced by precipitating Fe(OH)_3_, which instead lowers the concentrations of Fe^3+^ ions (Nidheesh, 2015). At high pH, H_2_O_2_ is also unstable and consumed by decomposition (Galbács and Csányi, 1983). Also, the redox potential for HO^•^ is reduced at higher pH which makes it less effective in a reaction (Velichkova et al., 2017).

As the optimum ratio between H_2_O_2_ and Fe^2+^ depends on several factors, including pH, it is important to find the optimal H_2_O_2_/Fe^2+^ ratio for the specific conditions (Rubio-Clemente et al., 2019). For example, the optimal ratio for oxidation of benzo(a)pyrene in natural water was found to be high; H_2_O_2_/Fe^2+^  = 39 (Rubio-Clemente et al., 2019), while in another study the optimum was H_2_O_2_/Fe^2+^  = 10 (Homem et al., 2009). In this study, see Fig. 2 and Table 4, the optimal H_2_O_2_/Fe^2+^ for the spiked sediment was 15 (FS(5;15)), while for the original sample the best TBT removal was achieved when no additional Fe^2+^ was added (FO(5;0)). The results of a classic Fenton oxidation process on spiked contaminated sediments showed a degradation after 1 h for MBT, DBT, and TBT, of 30, 68, and 81%, respectively (Brosillon et al., 2016). Here, 5.9 M H_2_O_2_ and 0.18 M Fe^2+^ were used, resulting in a ratio of ~ 33, which is higher than attempted in this study. This suggests that the optimal H_2_O_2_/Fe^2+^ ratio is dependent on multiple factors, including grain size and the presence of other reactive compounds in the sediment. For both original and spiked samples, the relation between added Fe and the percentage of degradation of the organotin compounds seems to be of less importance than the amount of added H_2_O_2_ (Table 4). The lesser effect of adding Fe may be due to the sediment already having a sufficient Fe content to reach an effective Fenton reaction, making the addition of extra Fe superfluous. However, an excess of Fe^2+^ could stop the Fenton reaction, as earlier discussed (Neyens and Baeyens, 2003; Flotron et al., 2005). Additionally, a high Fe content could have a toxic environmental effect (Krishnan et al., 2017).

2 $${\mathrm{H}}_{2}{\mathrm{O}}_{2}+{\mathrm{Fe}}^{2+}\to {\mathrm{Fe}}^{3+}+{\mathrm{OH}}^{-}+ {\mathrm{HO}}^{\bullet }$$

3 $${\mathrm{Fe}}^{3+}+{\mathrm{H}}_{2}{\mathrm{O}}_{2}\to {\mathrm{Fe}}^{2+}+{\mathrm{HO}}_{{2}^{\bullet }}+ {\mathrm{HO}}^{\bullet }$$

4 $${\mathrm{HO}}^{\bullet }+{\mathrm{H}}_{2}{\mathrm{O}}_{2}\to {\mathrm{HO}}_{{2}^{\bullet }}+{\mathrm{H}}_{2}\mathrm{O}$$

5 $${\mathrm{HO}}^{\bullet }+{\mathrm{Fe}}^{2+}\to {\mathrm{Fe}}^{3+}+{\mathrm{OH}}^{-}$$

6 $${\mathrm{Fe}}^{3+}+{\mathrm{HO}}_{{2}^{\bullet }}\to {\mathrm{Fe}}^{2+}+{\mathrm{O}}_{2}{\mathrm{H}}^{+}$$

7 $${\mathrm{Fe}}^{2+}+{\mathrm{HO}}_{{2}^{\bullet }}+{\mathrm{H}}^{+}\to {\mathrm{Fe}}^{3+}+{\mathrm{H}}_{2}{\mathrm{O}}_{2}$$

8 $${\mathrm{HO}}_{{2}^{\bullet }}+{\mathrm{HO}}_{{2}^{\bullet }}\to {\mathrm{H}}_{2}{\mathrm{O}}_{2}+ {\mathrm{O}}_{2}$$

In the leachates, most analyzed TBT concentrations were ≤ 1% of the initial TBT sediment content for both spiked and original samples (Supplementary information Table A2). This shows that TBT in the sediment and leachates is effectively degraded and not simply leached out into the aqueous phase. However, for one sample, FS(0.2;7), with a relatively low amount of added H_2_O_2_, 7% of the initial TBT content in the sediment remained in the water phase, highlighting the importance of the presence of H_2_O_2_ for efficient reduction. The leachates were all higher than the EU environmental quality standard for TBT in surface water (0.2 ng/L) (European Commission, 2005), which means that the leachates need further treatment to reach that concentration. The sediment had a pH of < 3 after treatment. This low pH would require further treatment to adjust it to a level appropriate for management practices. In addition, there is a risk that toxic byproducts will form during the oxidation of organic compounds in sediments using Fenton’s reagents (Pignatello et al., 2006).

The results indicate that Fenton’s reagent provides a feasible method for the degradation of TBT, DBT, and MBT. Future studies may be needed to investigate parallel reactions, e.g., competing radical reactions and parameters affecting the degradation in the sediment matrix. To further reduce the TBT content and risks posed by the sediment, sequential treatment could be implemented (Yan et al., 2015). For instance, this procedure could be repeated or coupled with sediment washing using ultra-pure water (Norén et al., 2021).

#### Electrochemical experiments

Boron-doped diamond (BDD) anodes are known to provide a very effective production of hydroxyl radicals in aqueous solutions (Moreira et al., 2017). The reaction is shown in Eq. 9, and the electrical current is proportional to the generation rate of hydroxyl radicals at the anode surface (Kraft, 2007; Brosillon et al., 2016).

9 $${\mathrm{H}}_{2}\mathrm{O}\to {\mathrm{HO}}^{\bullet }+{\mathrm{e}}^{-}+{\mathrm{H}}^{+}$$

**Fig. 3:**
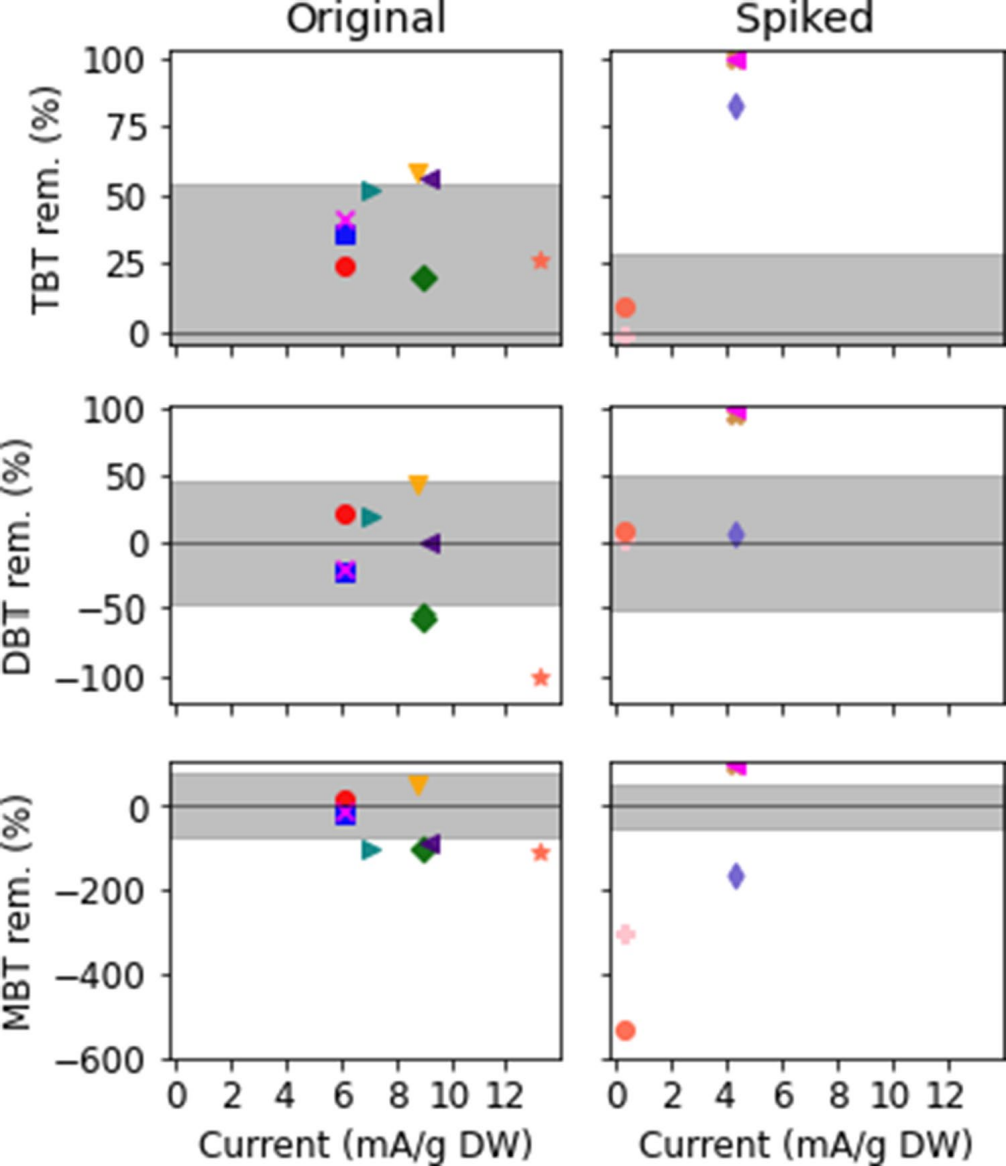
Visualization of the result presented in Table 5. The TBT, DBT, and MBT removal (%) in original (graphs on the left) and spiked sediment (graphs on the right) are shown in relation to the average applied current per sediment dry weight (mA/g DW). Each experiment is given a unique color and marker to highlight which results (TBT, DBT, and MBT) belong together. The shaded areas are marking the interval in which an increase or reduction is significant (p < 0.05, one-sample t test) considering variations in initial content

A higher amount of hydroxyl radicals generated per mass of sediment should lead to improved degradation of TBT. This is clearly seen for the spiked sample in this study, while the results for the original samples are more varied (Table 5 and Fig. 3). In Table 5, the results from the electrochemical experiments are presented, and in Fig. 3 the results in terms of removal efficiency are presented in relation to the organotin content variation in the untreated sediment (*p* < 0.05, one-sample *t* test). With spiked sediment, ~ 100% TBT removal was observed at 4.4 mA/g DW (120 mA/cm^2^), reclassifying the sediment from *very high* to *medium high content* for TBT and *low content* for DBT and MBT according to the Swedish marine sediment classification for organic pollutants (SGU, 2017). However, not all the TBT had been completely degraded. Approximately 1–9% of the TBT was found in the leachate of the spiked samples (Supplementary information Table A3), and increased content of MBT in the sediment indicated that some of the TBT had only degraded partially. In the original sediment, the maximum TBT removal of 58% was reached at 8.8 mA/g DW. However, at 9 and 13.2 mA/g DW, the TBT reduction was only ~ 25%. This could be explained by the natural variation in the sample. However, it was observed that the DBT and MBT contents, which both have a lower confidence interval and less variation in content than TBT, increased more than the calculated content variation, which indicates that TBT degradation occurred in these samples as well. The lower TBT removal efficiency in the original samples compared to the spiked samples is discussed earlier in chapter Characterization of sediment and seen in the Fenton experiments as well. The 58% TBT removal efficiency was sufficient to reclassify the sediment from *very high TBT content* to *high TBT content* according to the Swedish marine sediment classification for organic pollutants (SGU, 2017). DBT was also reclassified as *high content* and MBT as *medium high content*.

**Table 5:** The content of TBT, DBT, and MBT [µg/kg DW] in the residual sediment after electrochemical treatment

Experiment	TBT µg/kg DW	DBT µg/kg DW	MBT µg/kg DW
ES(5 V)	25,000	2400	2900
ES(21 V)	1.2	< 1	< 1
ES(23 V)	97	130	19
EO(0 V)	206	26	4.7
EO(15 V)*	103	42	20
EO(19 V)	130	61	39
EO(20 V)	66	23	9.3
EO(24 V)	120	78	40
EO(19 V) 400 g	76	32	38
EO(24 V) 400 g	68	40	36

^*^ Average of triplicate experiment with a standard deviation for TBT of 13 µg/kg DW, DBT of 9.7 µg/kg DW, and MBT of 3.6 µg/kg DW

The test EO(0 V), which used the original sample and no applied current, showed that TBT did leach out into the water (8.6 ng/L) and the TBT content in the sediment did not decrease. For all experiments using original samples, < 0.2% of the initial TBT content was found in the leachate, while a reduction in the TBT content in sediment was seen after treatment. This indicates that already with a low applied current, the TBT that leached out into the water phase was degraded. This is consistent with other studies, demonstrating that TBT in the water phase is more easily degraded than in the sediment phase (e.g., Stewart and de Mora, 1990; Ayanda et al., 2012; Brosillon et al., 2014). However, the analyzed leachates exceeded the European quality standard for the protection of the pelagic community, 0.2 ng/L TBT (Supplementary information Table A3) (European Commission, 2005). This indicates that although the TBT concentration was lower after treatment, the leachate needs to be further treated to not pose a threat to the environment. One option is to perform additional electrolysis on the leachates. In this project, the TBT degradation in saline and slightly saline water samples, spiked with TBTCl (1 µg/L) respectively, was studied. For both tests, TBT was degraded together with DBT and MBT to concentrations below the limit of quantification (TBT < 20 ng/L, DBT and MBT < 100 ng/L) (Supplementary information Table A4). This was achieved with a current density of 0.4 A/cm^2^ for the slightly saline water and 3.4 A/cm^2^ for the saline water. This difference in current density is due to the differences in salt concentrations. In the corresponding spiked blank samples (no current applied), the TBT concentrations were 650 ng/L in the saline solution and 619 ng/L in the slightly saline solution. This demonstrates the potential of using electrolysis for TBT contaminated water but also highlights that it is more difficult to treat sediment in comparison to water.

**Fig. 4:**
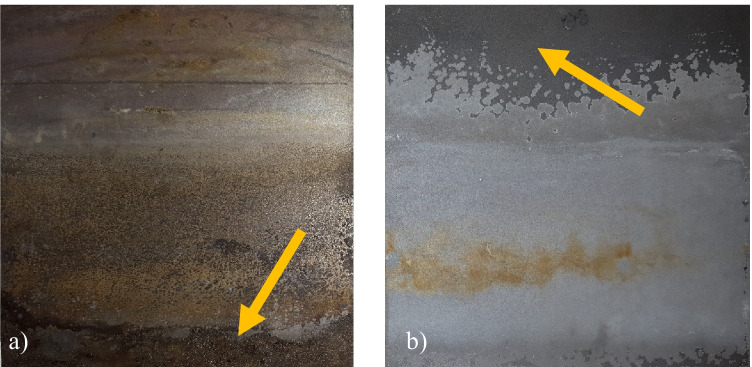
**a)** Titanium cathode with salt build-up. The build-up is more pronounced on the lower part of the cathode (see arrow). **b)** Delaminated niobium boron-doped diamond anode (Nb/BDD). On the upper part of the anode, the original darker gray surface is still visible (see arrow)

Two previous studies on electrochemical TBT oxidation with different experimental setups have observed higher removal efficiencies than this study (83–94%) at lower current density (Arevalo et al., 2004; Stichnothe et al., 2005). In these studies, the initial TBT content was similar to the original content in this study, ~ 200 g/kg DW. However, the type of electrodes used was different (Ti/IrO_2_ anode and steel cathode). IrO_2_ has a lower oxygen evolution potential than BDD and is, therefore, less likely to produce hydroxyl radicals as oxidants. Instead, reactive chlorine species can be produced from chloride ions (Moreira et al., 2017; Lu et al., 2019). Arevalo and Calmano (2007) compared TBT removal using either Ti/IrO_2_ or BDD anodes and did not observe any significant differences for the treatment of contaminated saline water. This implies that not only does the current density affect the TBT removal, but also a lot of different factors, such as the electrode surfaces (type and area), the occurrence of TBT in the sediment (spiked using a pure chemical or introduced by sedimented paint flakes), treatment time, particle size, and amount of the sediment treated. Additionally, the resistance for delamination of the anode may vary depending on the material used. After continuously treating sediment, the anode displayed a change in surface color (Fig. 4a). This could be an indication of delamination of the Nb/BDD electrode (Lu et al., 2019). Delamination could result in less effective treatment as time passes and the delaminated surface area increases. In this study, spiked sediments were treated before the original sediment was tested. This means that the original sediment samples could have reached higher organotin degradation if delamination did not occur. However, in a full-scale application, it is important to be aware of the delamination effect, as this will be performed over a longer period. Another problem related to the electrochemical treatment is the corrosion that occurred over time on both the crocodile clips attached to the electrodes (especially on the anode), which could spread to the cables connecting the electrodes to the voltmeter. Over time, build-up started to appear on the cathode (Fig. 4b). Metal analysis of the build-up on the cathode showed that it consisted mainly of Ca (60%) and Fe (28%). Precipitates are often observed in electrochemical systems, as the pH may rise near the cathode. This occurs because cathodic reactions such as oxygen reduction (Eq. 10) and hydrogen evolution (Eq. 11) consume hydrogen ions (Modin and Fukushi, 2012). The salt build-up could result in reduced performance and increased power consumption because the conductivity and active surface area of the cathode are reduced.

10 $${\mathrm{O}}_{2}+4{\mathrm{H}}^{+}+4{\mathrm{e}}^{-}\to 2{\mathrm{H}}_{2}\mathrm{O}$$

11 $$2{\mathrm{H}}^{+}+2{\mathrm{e}}^{-}\to {\mathrm{H}}_{2}$$

After treatment, the sediment had a distinct chlorine smell. BDD anodes have previously been shown to oxidize Cl^−^ ions into chlorine gas (Eq. 12) (Polcaro et al., 2009). Chlorine gas (Cl_2_) reacts with water and forms hypochlorous acid and hypochlorite ions depending on the pH (Eqs. 13–14).

12 $$2{\mathrm{Cl}}^{-}\to {\mathrm{Cl}}_{2}+2{\mathrm{e}}^{-}$$

13 $${\mathrm{Cl}}_{2}+{\mathrm{H}}_{2}\mathrm{O}\to \mathrm{HClO}+{\mathrm{H}}^{+}+{\mathrm{Cl}}^{-}$$

14 $$\mathrm{HClO}\leftrightarrow {\mathrm{ClO}}^{-}+{\mathrm{H}}^{+}$$

Chlorine species could contribute to TBT oxidation (Arevalo and Calmano, 2007) but are less effective for the treatment of chlorinated organic compounds (Murugananthan et al., 2008). The chlorine species are likely less efficient than hydroxyl radicals, which have a reduction potential of 2.8 V (Wang and Zhang, 2018), while free Cl_2_ has a reduction potential of 1.358 V (Kaczur, 1996). However, in the presence of organic compounds, they can also lead to the formation of toxic halogenated organics, which has been observed in the electrochemical treatment of wastewater (Jasper et al., 2017). Despite lower sediment toxicity after electrochemical treatment, there may be a need for further treatment to remove any remaining oxidative species (Stichnothe et al., 2005; Arevalo and Calmano, 2007). An advantage of the electrochemical treatment was that the sediment’s pH was relatively unaffected by the treatment. From a starting pH of 8.2, it was reduced to 7.4 by the electrolysis treatment. This means that no pH adjustment is necessary before sediment utilization or disposal.

##### Metal removal

**Fig. 5:**
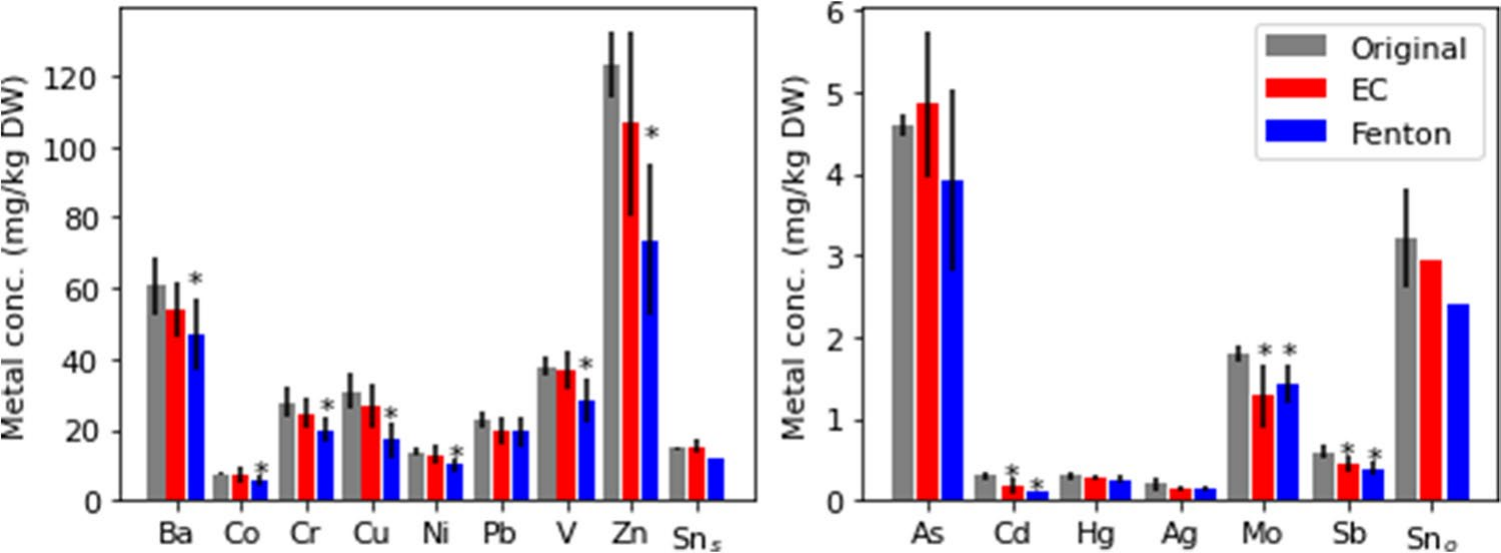
Average content and standard deviation for metals in original sediment (gray bars) and sediment treated electrochemically (EC) (red bars) and with Fenton’s reagent (Fenton) (blue bars). The result is presented in two graphs with different scales due to the large variation in metal content in the sediments, where metals found in higher content are presented in the left graph and metals found in lower contents are presented in the right graph. The tin contents in the sediment before and after treatment are presented for spiked samples (Sn_s_) and original samples (Sn_o_) due to different starting values. * denotes values that are significantly lower than the original content according to Welch’s t test (p < 0.05)

The metal contents were analyzed in and compared between, the original and spiked samples, as well as the treated sediments, as the metal contents are not affected by the addition of TBT, except concerning Sn (Supplementary information Table A1). The sediment contents of most analyzed metals were reduced after treating the sediment with both Fenton’s reagent and electrochemical oxidation (Fig. 5). Metals were removed to a somewhat larger extent from the Fenton-treated sediments compared to the electrochemically treated sediments. This is probably due to the low pH (~ 2.0–3.2) in the Fenton process (Gambrell et al., 1991; Zang et al., 2017) causing a metal release in the leachates, but it may also be explained by the degradation of organic matter in the sediments (Kalmykova et al., 2008; Wang and Mulligan, 2009; Mecozzi et al., 2011). Metals (e.g., Cu and Cd) bound to humic acids and other organic acids in sediments through complexation are released if the acids are degraded during the oxidation or protonated due to the low pH. The highest removal percentage of metals in Fenton-treated sediment was for Cd (56%), Cu (45%), and Zn (40%) (Table 6). The high removal of Cd may be due to its tendency to bind to sulfate ions (Rao et al., 2011) which are altered during electrolysis (e.g., SO_4_^2−^  → S_2_O_8_^2−^) (Silva et al., 2021) which could result in a potential release. Additionally, under oxidative settings Cd tends to transform into an ionic form (Zito, 2020). Also, in an earlier study where a sequential leaching test of sediment sampled close to the site investigated in this study, it was shown that Cd in particular binds to the absorbed and exchangeable fraction in the sediment (63% of the Cd) (Norén et. al., 2021), which could be released due to the oxidation. The low content of Cd could as well play a role, as the uncertainties during measurements could make the percentage appear higher.

**Table 6:** Removal rates of organotins and metals from sediment presented together with estimated treatment cost and management aspects. For the organotin removal, the experimental setup for the highest TBT reduction is presented. For the metals, the average and the relative standard deviation are presented for Fenton’s reagent (n = 7) and electrochemical treatment (n = 6)

	**Electrochemical treatment**	**Fenton’s reagent**
**Removal (%)**		
**TBT**	58	64
**DBT**	43	22
**MBT**	51	63
**Ag**	29 ± 18	32 ± 23
**Cd**	32 ± 55	56 ± 4
**Cu**	13 ± 23	45 ± 28
**Ni**	6 ± 22	27 ± 17
**Pb**	14 ± 21	15 ± 23
**Zn**	13 ± 25	40 ± 29
**Indicative cost (USD/tonne)**	900 (+ 600^*^)	4100
**Work requirements**	Oxidation proof materials (e.g., containers)	Oxidation proof materials (e.g., containers)
	Safety equipment	Safety equipment
	Proper ventilation	Proper ventilation
		Safe chemical storage
**Residue**	Neutral pH	Acidic pH
	Treatment does not alter sediment grain size	Finer grained sediment after treatment
	Further treatment might be required	Further treatment might be required

^*^Initial electrode cost regardless of mass treated (as service life time is unknown)

Metal ions can be recovered from solutions by electrochemical reduction on a cathode. However, metal ions are mainly mobilized at acidic pH, as discussed in the previous paragraph. After electrochemical treatment, the pH of the sediment was neutral (pH ~ 7.4) and the leachate was alkaline (pH ~ 9.0). Despite the neutral pH, the electrolysis resulted in a moderate removal percentage for Cd (32%) and Ag (29%). On the other hand, previous studies on sediment (Arevalo et al., 2004; Stichnothe et al., 2005) reported that the metal content is unaffected by the electrolysis. However, a higher current was applied in this study, demonstrating the importance and potential of having a high applied current if metal removal is desired. If acid was added to the sediment–water slurry, the metal recovery rate would probably increase due to decreased pH (Pedersen et al., 2017). By using a bioelectrochemical system (BES), a higher metal recovery could potentially be achieved with less energy, even where the metal concentration in the leachate is low (Modin et al., 2012). Another option could be to combine Fenton’s reagent with (BES), so-called bio-electron-Fenton (Wang et al., 2019), or combine electrolysis and Fenton with heterogeneous catalysis (Poza-Nogueiras et al., 2018), or even combine Fenton reagent with photoelectrocatalysis (Garcia-Segura and Brillas, 2017).

Cadmium was reduced to background levels for both treated sediments, according to the Norwegian environmental quality standards for sediment (Miljødirektoratet, 2016; Direktoratsgruppen vanndirektivet, 2018). For Fenton-treated sediment, Cu was also lowered to background levels, at which it does not pose a risk for sporadic adverse effects to occur. The release of several metals from the sediment shows the potential for metal recovery from the leachates and the cathode. However, as the major pollutant in the sediments studied here is TBT, the metal contents are generally low. For Zn, present in the highest content and with a high release, the resulting concentration accounts for 8 mg/L if an L/S ratio of 6.25 (as in the Fenton experiments) is used, which is too low for an economically viable recovery. However, for sediments polluted with higher metal content, recovery using Fenton’s reagent and/or electrolysis is interesting for further studies, especially if the sediment also contains TBT, as this is efficiently degraded using these treatments.

### Method comparison

In Table 6, TBT, DBT, and MBT removal rates for both methods are presented with metals selected for their high concentrations relative to guideline values (see chapter Characterization of sediment) or for their monetary values (Norén et al., 2020). Removal rates were found to be higher in Fenton samples for TBT, MBT, and metals, while electrolysis was found to more efficiently remove DBT. However, a comparison of the methods also needs to consider drawbacks. Fenton results in residual sediments with low pH, which requires further treatment before potential reuse. Potential anode delamination and generation of Cl_2_ (g) are potential drawbacks of electrolysis. Both methods require proper safety equipment for the processes as well as personal safety equipment for workers. Further, both methods require durable equipment suitable for strong oxidization as well as the management of fumes that form during the treatment. Using Fenton’s reagent would require chemical management as the method include the addition of acid and H_2_O_2_, unlike the electrochemical treatment. Both methods might also require further treatment of the sediment, e.g., leachate treatment for both methods and sediment pH neutralization for Fenton, which is associated with additional costs. Based on removal rates and potential drawbacks, we suggest that Fenton is more suitable for heavily polluted sediment, as valuable metals could potentially be recovered from the leachate through electrolysis or chemical precipitation in an additional step. For less metal-polluted sediment, electrolysis could be sufficient to reduce the TBT content as the sediment residue is less affected by the treatment. Additionally, both treatment methods in this study need to be adjusted and optimized before full-scale application, as well as to perform a more extensive cost and risk estimation.

Simplified cost estimations for the treatment methods have been done based on the experimental setup used for the highest TBT removal for both methods (Table 6). For Fenton, the cost of laboratory quality H_2_O_2_ is ~ 9.7 USD/L (Fisher Scientific n.d.), and as no Fe^2+^ is added in the setup reaching the highest TBT removal, the Fenton treatment cost is estimated to be about ~ 4.1 USD/kg sediment. However, the cost for chemicals might be reduced if large quantities are purchased and lower quality is used in full-scale applications. For electrolysis, the cost refers to the purchase of electrodes. The BDD/Nb anode used in this study costs approximately 600 USD for a 10 × 10 cm electrode, and in comparison to BDD/Nb, the cost for the Ti cathode is neglectable. The size is likely to be increased for full-scale operation and the electrodes regularly change with time. It is unknown how long a BDD/Nb anode would last for the treatment of sediment. In a study where water was treated at a significantly higher current density, 1 A/cm^2^, it started to show performance reduction after about 500 h (Lu et al., 2019). In this study, less current density was applied for the experiment with the highest TBT removal (0.001 A/cm^2^). This should result in longer service life for the anode. However, it is likely that treating sediment is considerably tougher on the electrodes in comparison to water samples, as sediment grains could cause mechanical grinding and lower the anode service life. However, it is possible to recoat the electrodes instead of buying new ones. For simplicity, it is assumed that a pair of electrodes could be used to treat a tonne of sediment in the examples in Table 6. In comparison to the cost of electrodes, the electrochemical treatment cost is considerably low. The sustainability and electricity cost depends on the source of energy (Vocciante et al., 2021), but for simplicity, the average price in Sweden is used, 0.05 USD/kWh, and of which ~ 50% have a renewable origin (water and wind) (The Swedish Consumer Energy Markets Bureau n.d.). This results in an electricity cost of 900 USD to treat 1 tonne sediment, which is negligible compared to the cost for electrodes. As discussed for Fenton, electrode prices may be reduced if many electrodes are bought. In these simplified assumptions, it is seen that for small quantities of sediment (< 0.2 tonnes) Fenton’s reagent is cheaper than electrochemical degradation due to the high initial cost of electrodes. For sediment masses > 0.2 tonnes, electrolysis might be a more economic option. However, when scaling up the methods to treat tonnes of masses, the true cost would most likely differ in comparison to the cost for laboratory-scale experiments.

## Conclusions

This study investigated whether oxidation by either Fenton’s reagent or electrochemical treatment could remove organotin compounds and metals from contaminated dredged marine sediment. Both methods were found to be effective in reducing the TBT content in the sediments, showing that the methods have the potential to be scaled up and possibly used on-site to treat contaminated sediments. For spiked sediments, a removal rate of > 98% TBT was achieved, whereas the removal efficiencies were lower for the original sediment (64% Fenton and 58% electrolysis). The TBT concentration in spiked water was reduced down to < 20 ng/L, demonstrating that TBT sorbed to sediment is more difficult to treat than TBT solved in water. For the electrochemical treatment, TBT removal was positively correlated with the applied current per mass unit sediment. For Fenton’s reagent, the added amount of H_2_O_2_ was positively correlated with TBT removal, while the addition of Fe^2+^ seems to be less important for the removal, probably due to the natural Fe^2+^ content in the sediment. Both methods reduced metal contents in the sediments, but Fenton-treated sediment showed a slightly higher metal reduction, in particular for Cd, Cu, and Zn. The low pH after Fenton treatment implies that additional measures are required to handle the sediment, whether it is due for disposal or reuse, e.g., as filling materials or in construction. The pH of the electrochemically treated sediment, on the other hand, was relatively unaffected compared to the original samples, why the electrochemically treated sediment may be more suitable for direct use. Fenton’s reagent may be a suitable option for treatment of heavily metal-polluted sediment, but for treatment of large quantities of TBT contaminated sediment electrochemical degradation could be a more appropriate option. To sum up, this study discusses two promising methods for degrading organotin compounds in sediments, but before application, at full scale, further studies on the occurring reactions and potential production of toxic byproducts are recommended, as well as optimizing the contaminant removal. Potentially, these methods could be combined with other advanced oxidation methods to achieve an even more efficient TBT degradation and higher metal recovery. The findings from this study may be useful for sediment managers who need to reduce TBT and metal content in sediments.

## Supplementary Information

Below is the link to the electronic supplementary material.

Supplementary file1 (PDF 741 KB)

## Data Availability

The datasets used and/or analyzed during the current study are available from the corresponding author on reasonable request.
