# Supplementary material for: Removal of organotin compounds and metals from Swedish marine sediment using Fenton’s reagent and electrochemical treatment
Source: Environ Sci Pollut Res Int. 2022 Jan 5;29(19):27988–8004. doi: 10.1007/s11356-021-17554-8 (PMC8993779; doi:10.1007/s11356-021-17554-8)
Supplement: Supplementary file 1 — Supplementary file1 (PDF 741 KB) [file 11356_2021_17554_MOESM1_ESM.pdf]

Supplementary information for

# Removal of organotin compounds and metals from Swedish marine sediment using Fenton's reagent and electrochemical treatment

Anna Norén[a,*], Célia Lointier[a], Oskar Modin[a], Ann-Margret Strömvall[a], Sebastien Rauch[a], Yvonne Andersson-Sköld[c,d], Karin Karlfeldt Fedje[a,b]

[a] *Department of Architecture and Civil Engineering, Division of Water Environment Technology, Chalmers University of Technology, SE-412 96 Gothenburg, Sweden*

[b] *Recycling and Waste Management, Renova AB, Box 156, SE-401 22 Gothenburg, Sweden*

[c] *Swedish National Road and Transport Research Institute (VTI), Box 8072, SE-402 78 Gothenburg, Sweden*

[d] *Department of Architecture and Civil Engineering, Division of Geology and geotechnics, Chalmers University of Technology, SE-412 96 Gothenburg, Sweden*

[*] *Corresponding author, anna.noren@chalmers.se*

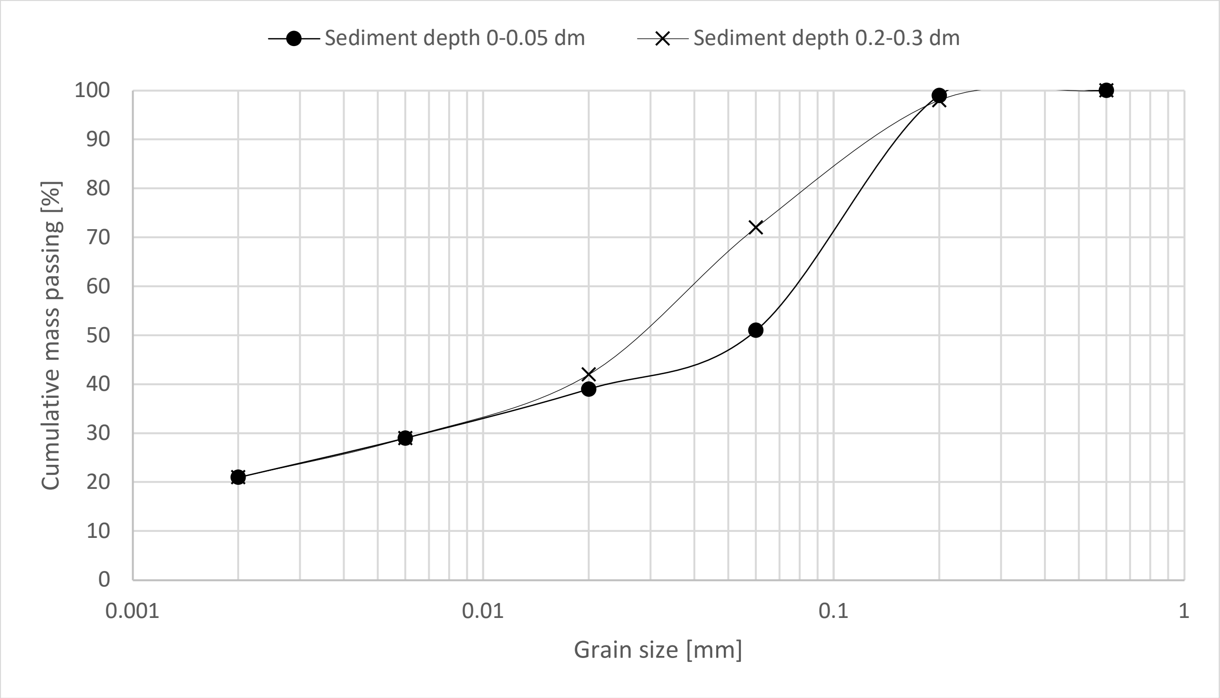


*Figure A1 Sem-log cumulative grain size distribution curve for two sediment samples sampled at different depth before the dredging.*

*Table A1 Metal content [mg/kg DW] and standard deviation (STD) in original, electrochemically treated (Electro) and Fenton's reagent treated (Fenton) sediment. *Fe was only analyzed in spiked samples.*

| Metals [mg/kg DW] | Original samples | | Electro | | Fenton | |
|---|---|---|---|---|---|---|
| | Average | STD | Average | STD | Average | STD |
| As | 4.6 | 0.1 | 4.9 | 0.9 | 3.9 | 1.1 |
| Ba | 61.0 | 8.1 | 54.0 | 7.8 | 47.0 | 9.9 |
| Cd | 0.3 | 0.0 | 0.2 | 0.1 | 0.1 | 0.0 |
| Co | 7.3 | 0.4 | 7.6 | 2.3 | 5.7 | 1.5 |
| Cr | 28.0 | 4.0 | 25.0 | 4.4 | 20.0 | 3.5 |
| Cu | 31.0 | 4.9 | 27.0 | 6.1 | 17.0 | 4.8 |
| Fe* | 17,500 | 2,800 | - | - | - | - |
| Hg | 0.3 | 0.0 | 0.3 | 0.0 | 0.3 | 0.0 |
| Ni | 14.0 | 0.9 | 13.0 | 2.9 | 10.0 | 1.7 |
| Pb | 23.0 | 2.1 | 20.0 | 4.1 | 19.0 | 4.5 |
| V | 38.0 | 2.5 | 37.0 | 5.8 | 28.0 | 6.5 |
| Zn | 120.0 | 9.3 | 110.0 | 26.0 | 74.0 | 21.0 |
| Ag | 0.2 | 0.1 | 0.2 | 0.0 | 0.2 | 0.0 |
| Mo | 1.8 | 0.1 | 1.3 | 0.4 | 1.4 | 0.2 |
| Sb | 0.6 | 0.1 | 0.4 | 0.1 | 0.4 | 0.1 |

*Table A2 TBT, DBT and MBT concentrations [ng/L] in leachates after treatment with Fenton's reagent.*

| Experiment | TBT ng/L | DBT ng/L | MBT ng/L |
|---|---|---|---|
| FS(0.01;4) | 880 | 3,000 | 1,400 |
| FS(0.2;7) | 28,000 | 5,700 | 1,700 |
| FS(1;2) | 320 | 360 | 220 |
| FS(5;5) | 5,100 | 12,000 | 810 |
| FS(7;5) | 920 | 4,300 | 1,500 |

*Table A3 TBT, DBT and MBT concentrations [ng/L] in leachates after electrochemical treatment.*

| Experiment | TBT ng/L | DBT ng/L | MBT ng/L |
|---|---|---|---|
| ES(5V) | 2,500 | 690 | 3,300 |
| ES(21V) | 24,000 | 25,000 | 23,000 |
| ES(23V) | 36,000 | 52,000 | 42,000 |
| EO(0V) | 8.6 | 2 | 3.9 |
| EO(15V) | 11 | 16 | 8.6 |
| EO(19V) | 11 | 4.1 | 2.2 |
| EO(24V) | 1.7 | 26 | 43 |

*Table A4 TBT, DBT and MBT concentrations [ng/L] in spiked water after electrochemical treatment and in blanks (not electrolyzed reference samples).*

| Experiment | TBT ng/L | DBT ng/L | MBT ng/L |
|---|---|---|---|
| Saline water | <20 | <100 | <100 |
| Saline water blank | 650 | <100 | <100 |
| Slightly saline water | <20 | <100 | <100 |
| Slightly saline water blank | 619 | <100 | <100 |
